# Inhibition of SIRT1 deacetylase and p53 activation uncouples the anti-inflammatory and chemopreventive actions of NSAIDs

**DOI:** 10.1038/s41416-018-0372-7

**Published:** 2019-02-11

**Authors:** Giulia Dell’Omo, Daniela Crescenti, Cristina Vantaggiato, Chiara Parravicini, Aurora Paola Borroni, Nicoletta Rizzi, Mariangela Garofalo, Andrea Pinto, Camilla Recordati, Eugenio Scanziani, Fabio Domenico Bassi, Giancarlo Pruneri, Paola Conti, Ivano Eberini, Adriana Maggi, Paolo Ciana

**Affiliations:** 1 Department of Oncology and Hemato-Oncology, University of Milan, 20133 Milan, Italy; 2 Department of Pharmacological and Biomolecular Sciences and Center of Excellence on Neurodegenerative Diseases, University of Milan, 20133 Milan, Italy; 3 Department of Pharmaceutical Sciences, University of Milan, 20133 Milan, Italy; 4 Mouse & Animal Pathology Lab, Fondazione Filarete, 20139 Milan, Italy; 5 Division of Breast Surgery, European Institute of Oncology Milan, 20141 Milan, Italy; 6 Pathology and Laboratory Medicine, Fondazione IRCCS Istituto Nazionale dei Tumori (INT), 20133 Milan, Italy

**Keywords:** Target identification, Structure-based drug design, Cancer prevention, Mechanism of action, Breast cancer

## Abstract

**Background:**

Nonsteroidal anti-inflammatory drugs (NSAIDs) have been proposed as chemopreventive agents for many tumours; however, the mechanism responsible for their anti-neoplastic activity remains elusive and the side effects due to cyclooxygenase (COX) inhibition prevent this clinical application.

**Methods:**

Molecular biology, in silico, cellular and in vivo tools, including innovative in vivo imaging and classical biochemical assays, were applied to identify and characterise the COX-independent anti-cancer mechanism of NSAIDs.

**Results:**

Here, we show that tumour-protective functions of NSAIDs and exisulind (a sulindac metabolite lacking anti-inflammatory activity) occur through a COX-independent mechanism. We demonstrate these NSAIDs counteract carcinogen-induced proliferation by inhibiting the sirtuin 1 (SIRT1) deacetylase activity, augmenting acetylation and activity of the tumour suppressor p53 and increasing the expression of the antiproliferative gene p21. These properties are shared by all NSAIDs except for ketoprofen lacking anti-cancer properties. The clinical interest of the mechanism identified is underlined by our finding that p53 is activated in mastectomy patients undergoing intraoperative ketorolac, a treatment associated with decreased relapse risk and increased survival.

**Conclusion:**

Our study, for the first-time, links NSAID chemopreventive activity with direct SIRT1 inhibition and activation of the p53/p21 anti-oncogenic pathway, suggesting a novel strategy for the design of tumour-protective drugs.

## Introduction

Cancer incidence is projected to increase worldwide particularly in view of the ever-increasing population lifespan;^1^ novel strategies for prevention are therefore needed to decrease the personal, social and economic burden of this disease. NSAIDs are among the most promising chemopreventive agents for different cancer types and could give an important contribution to the control of neoplasia development especially in high-risk groups;^2^ a number of in vivo preclinical data demonstrated the cancer-protective activity of these drugs^3–7^ and clinical studies assessed the protective effects of chronic or transient intraoperative treatments^8–12^ However, the use of NSAIDs in the treatment of a relatively healthy, at risk population is limited by the potentially serious adverse gastrointestinal and cardiovascular events. At present time, the lack of a full understanding of the mechanism of their anti-cancer effects has blocked the development of NSAIDs to be used safely in chemoprevention. Several mechanisms of action have been proposed to explain the anti-tumour properties of this structurally heterogeneous group of similarly acting compounds: the inhibition of COX enzymes, especially COX-2, the classical target of the anti-inflammatory actions of these drugs, was indicated as a major player to mediate their anti-cancer effects;^13^ in addition, several off-target actions were also proposed, including modulation of cancer-related pathways (e.g., WNT/β-catenin or cGMP/PDE), the activity of transcription factors (e.g., p53, PPARλ, PPARδ, SP1, NFkB, RXR) and enzymes (AMPK, carbonic anhydrase, Ca++ ATPase, MMPs) involved in carcinogenesis.^14^ The existence of COX-independent mechanisms responsible for the anti-tumour effect of these classes of drugs has been strongly supported by the discovery that NSAIDs enantiomers, derivatives or metabolites, which are not able to inhibit COX and don’t display anti-inflammatory properties, still retains anti-tumour activity in vitro and in vivo.^14^ In particular, the best-characterised molecule of this type is exisulind, a metabolite of sulindac, which was shown in clinical trials to induce adenoma regression in familial and sporadic adenomatous polyposis,^15,16^ and to inhibit the formation of multiple tumour types in several preclinical studies.^17^ Yet the picture emerging from these studies is still elusive and does not provide a molecular target alternative to COX as clearly accountable for the anti-neoplastic activity of NSAIDs. We here propose SIRT1 deacetylase as a novel common and direct target of NSAIDs and exisulind, which may provide a molecular explanation of the multiplicity of the COX-independent effects ascribed to NSAIDs and their metabolites/enantiomers/derivatives.

## Materials and Methods

### Reagents, antibodies and kits

Unless otherwise specified reagents were from Sigma-Aldrich. Antibodies used for immunoblot assay were acetylated p53 (K382) (#2570S, Cell Signaling), total p53 (Ab-7, Abcam) and SIRT1 antibody (05-1243, Merck Millipore). SIRT1 activity was measured in vitro according to the manufacturer’s protocol using a fluorimetric activity assay kit (ab156065, Abcam).

### Cell culture and treatments

MDA-MB-231 were purchased from the American Type Culture Collection (ATCC) and grown in RPMI 1640 medium (Life Technology) supplemented with 10% FBS (Sigma-Aldrich) and streptomycin-penicillin (50,000 IU plus 50 mg/l). Cells were treated with NSAIDs, exisulind, nicotinamide and DMSO as vehicle; compounds were appropriately diluted before treatment with cell culture medium from a stock of 27 mM dissolved in DMSO (Sigma Aldrich).

### Protein extracts and western blot

Protein extracts were obtained by suspending pellet of cells in lysis buffer (10 mM Tris-HCl, pH 7.4, 150 mM NaCl, 15% glycerol, 1% Triton-X-100, 1 mM sodium orthovanadate, 10 μg/ml leupeptin, 10 μg/ml aprotinin, 1 mM NaF, protease inhibitor cocktail and 1 mM PMSF), disrupting cell membranes by freezing and thawing and collecting supernatant after 30 min minifuge centrifugation at the maximal speed. 30 µg of protein extracts were separated in a PAGE and immunoblot assays were carried out using specific antibodies recognizing acetylated p53 (K382 residue) or total p53; immunoreactive bands were visualised with chemiluminescence by using ECL^TM^ Western Blotting Analysis System according to the manufacturer’s instructions (Amersham).

### P53 deacetylation assay

The SIRT1 deacetylase activity was tested on the K382 residue of native p53 present in the protein extract of MDA-MB-231 cells pre-treated with 20 μM etoposide. Briefly, rhSIRT1 (final dilution 1:60 ab156065 #6), 200 μM NAD and 300 μM inhibitors (NSAIDs or exilusind) were added to 15 μg protein extract of MDA-MB-231, incubated for 30 min at room temperature and stopped with the addition of Laemmli’s sample buffer. The entire reaction was loaded on a SDS-PAGE for immunoblot analysis; immunodetection was carried out using specific antibodies recognizing acetylated p53 (K382 residue) or total p53.

### Real-Time PCR

Real-Time PCR experiments were done as previously described.^18^ Templates were amplified using GoTaq® qPCR Master Mix (Promega) in a thermocycler (ABI Prism 7,000, Applied Biosystems). The following primers were used for each mouse gene: 36b4 forward 5′-ggcgacctggaagtccaact-3′, reverse 5′-ccatcagcaccacagccttc-3′; p21 forward 5′-gcctgaagactgtgatgg-3′, reverse 5′-gccctcagcaagagtaag-3′. Data were analysed using the ABI Prism 7,000 SDS Software and the 2^−ΔΔCt^ method. The levels of mRNA transcripts were normalised on the constitutively expressed gene 36b4.

### Stable transfection

MDA-MB-231 cell clones that stably express a siRNA targeting human SIRT1^19^ was generated by co-transfecting 1 µg pBABE SIRT1 siRNA (kindly provided by Prof. D.A. Sinclair) or 1 µg pBABE empty vector as a control, together with 1 µg renilla luciferase pRL-TK (E2241 Promega) and 0.1 µg pSV2Neo carrying the neomycin resistance for clone selection. Cells were transfected with Lipofectamine 2000 (Thermo Fisher) according to the manufacturer’s protocol. 48 h after transfection different dilution (from 1 × 10^6^ to 1 × 10^5^/petri) of cells were seeded in petri dishes. After 3 weeks in selection medium containing 600 µg/ml G418, single clones were picked and tested for Renilla luciferase expression with an enzymatic assay on protein extract carried out according to manufacturer’s protocol (Renilla-Glo Luciferase Assay System, Promega). Clones with higher levels of luciferase expression were further expanded and tested for SIRT1 expression by western blot analysis. Two clones displaying the lowest SIRT1 expression and two control clones (transfected with the empty vectors) were chosen for testing the effects of NSAIDs.

### Molecular modelling procedures

All the computational procedures were carried out by the Schrödinger Small-Molecule Drug Discovery Suite 2016-02. The crystallographic structure of the catalytic domain of human SIRT1 bound to NAD and to an EX-527 was downloaded from the RCSB PDB (code: 4I5I). The Schrödinger Protein Preparation Wizard was used for locating and fixing structural defects in SIRT1 structure and preparing it for use with Schrödinger Glide for molecular docking. Tested ligands were built by the Schrödinger Maestro Build Toolbar and prepared for docking by the Schrödinger Ligand Preparation. The molecular docking procedure was carried out by the Schrödinger Glide Docking in standard precision (SP) mode in order to evaluate the ability of the tested ligand to bind the SIRT1 catalytic domain. Only for ketoprofen and nicotinamide the docking procedure was carried out in extra precision (XP) mode in order to better sample the binding site and obtain more accurate poses. Molecular docking was run both on the *holo-*SIRT1 (w/ NAD) and on the *apo-*SIRT1 (w/o NAD). The top-scoring solution between the two poses (one obtained on the *apo*- and the other one on the *holo-*SIRT1) for each ligand was submitted to Schrödinger Prime MM-GBSA, which integrates molecular mechanics energies combined with the generalised Born and surface area continuum solvation^20^ in order to calculate ligand binding and ligand strain energies for a set of ligands and a single receptor.

### Animal experimentation

For the in vivo use, NSAIDs, exisulind and nicotinamide were dissolved in DMSO (270 mM stock solution); after appropriate dilution in water, they were given *per os* if not otherwise specified 15 mg/Kg/day and 3.75–7.5-15 mg/Kg/day exisulind. DMBA was dissolved in acetone (12 mM solution). 25 female repTOP*mito*IRE reporter mice (2–4 month-old)^21^ were divided in five groups and treated with NSAIDs, exisulind, nicotinamide or vehicle (DMSO) for 8 days. At day 5, mice were subjected to a single s.c. intra-glandular injection of 12 mM DMBA solution (left mammary gland) or acetone (vehicle, in the right mammary gland); at day 8, mice were sacrificed, the mammary glands explanted for ex vivo imaging and fixed for immunohistochemistry analysis or frozen for total RNA extraction.

### Ethical approval animal experimentation

All animal experimentation was carried out in accordance with the Guide for the Care and Use of Laboratory Animals in accordance with the European Guidelines for Animal Care and Use of Experimental Animals, approved by the Italian Ministry of the Research and University (MIUR) and controlled by the panel of experts of the Department of Pharmacological and Biomolecular Sciences (University of Milan, 20133 Milan, Italy). For the experiments before 2014 the MIUR authorisation was DM 295/2012-A dated 20.12.2012 n. 10/2012, afterward experiments were done under MIUR authorisation n. 611/2015 PR. All animal experimentation was carried out in the full observation of the Directive 2010/63/UE.

### Bioluminescence in vivo and ex vivo imaging

The procedure has been previously described.^22^ Briefly, anesthetised animals were i.p. injected with 65 mg/Kg D-Luciferin (beetle luciferin potassium salt, Promega) before each in vivo imaging session. After 15 min luciferin distribution, photon emission was measured over 5 min-exposure time using a CCD camera (Night Owl Imaging Unit, Berthold Technologies, Germany). After the last in vivo imaging acquisition, animals were sacrificed, the mammary glands were excised and placed in a light-tight chamber for the ex vivo measurement. Pseudocolor images representative of photon emissions were generated by a Night Owl LB981 image processor and transferred *via* video cable to a PCI frame grabber using WinLight32 software (Berthold Technologies); grayscale and pseudocolor images were finally merged using WinLight version 32 software (colour code from low to high photon emission: blue, green, red, yellow, and white). Light emission was expressed as integration of photon counts per time and per area unit (p/s/cm^2^/sr). Normalisation was performed using an external source of photons enabling to measure the instrumental efficiency of photon counting (Glowell Luxbiotech, Edinburgh, UK).

### Ethics approvals human material

All human tumour specimens were obtained in accordance with the Ethic Committee of the European Institute of Oncology, Milan, Italy and the main tumour features are listed in the Table S2.

### Statistical analysis

Data analyses were performed using GraphPad 5 Instat software® (GraphPad Prism Inc. San Diego, CA, USA), we have applied Student’s *t-*test, one-way ANOVA and two-way ANOVA analysis for determining statistical significance.

## Results

### NSAIDs and exisulind, but not ketoprofen, increase p53 acetylation at K382 residue

**Fig. 1:**
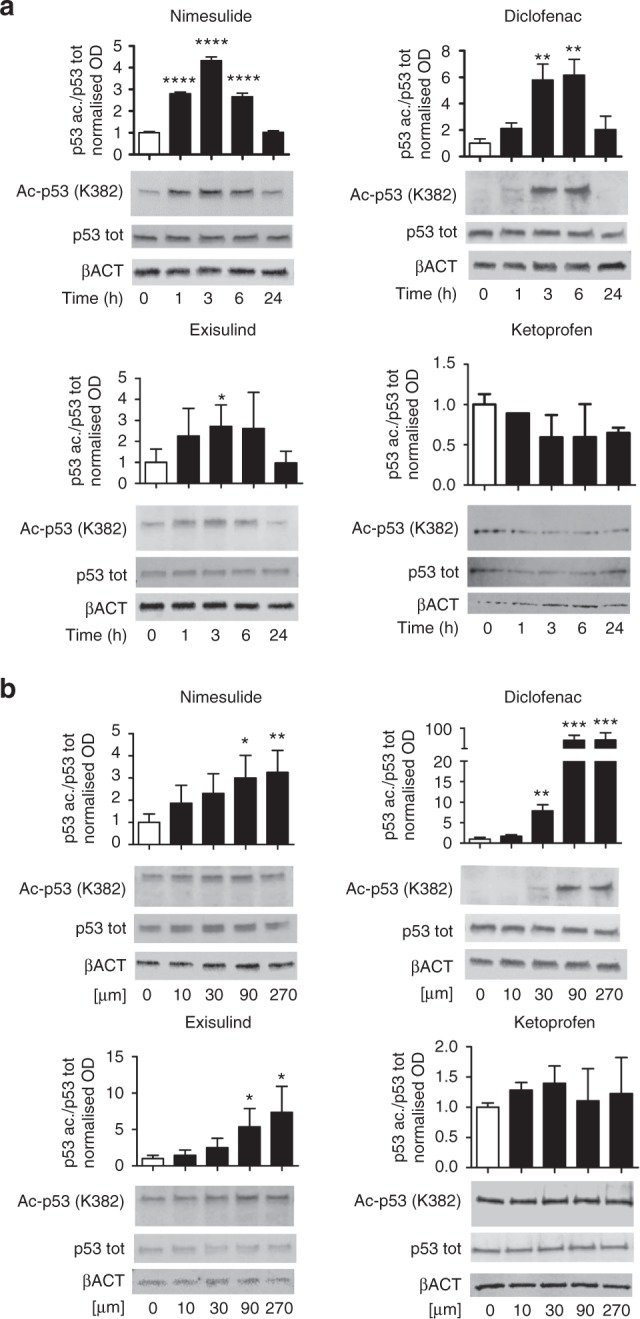
Treatment with nimesulide, diclofenac and exisulind, but not ketoprofen increase p53 acetylation at K382 residue. Data from more NSAIDs are shown in Fig. S1A. Immunoblot analysis was carried out using anti-acetyl (K382) p53 and anti-total p53 antibodies and protein extracts obtained from MDA-MB-231 cells in time course experiments (**a**) or after 3 h treatment with increasing concentrations (**b**) of the indicated NSAIDs. For the time course experiments, cells were treated with 90 μM for each compound. Data are represented as mean ± SEM. Bars in the graphs represents densitometry quantifications of the autoradiographic signals (acetylated p53 vs total p53); β-actin is reported as loading control. *P < 0.05 **P < 0.01 ***P < 0.001 ****P < 0.0001 versus the baseline level; *P*-values were calculated by one-way ANOVA followed by Bonferroni’s test

Several data demonstrated the anti-proliferative effect of NSAIDs in different tumour cell lines.^23,24^ In a previous study, Alfonso and collaborators^25^ showed that aspirin treatment promotes p53 acetylation at residue K382, increases expression of p21, a p53 target gene, and apoptosis in the MDA-MB-231 cell line. This led us to ask whether other NSAIDs with anti-proliferative activity in cultured cell lines shared the ability to increase p53 acetylation. The content of p53 isoforms (total and acetylated at the K382 residue) was therefore measured in MDA-MB-231 treated with 90 μM of nimesulide, diclofenac, and exisulind; for different lengths of time. Ketoprofen was also included in the study as a negative control since this drug has a poor anti-proliferative activity.^26^ All compounds, but ketoprofen, significantly increased p53 acetylation in a short time after exposure to the drug (3–6 h) (Fig. 1a) and such an effect was concentration-dependent (Fig. 1b); similar effect was observed for other NSAIDs suggesting a common mechanism of p53 acetylation for the tested molecules of this class of drugs (Figure S1A). Interestingly, p53 acetylation occurred mainly in the nucleus (Figure S1B) indicating that the NSAID-dependent mechanism selectively modifies the nuclear functions of the anti-oncogene.

### SIRT1 enzymatic activity is inhibited by several NSAIDs and exisulind, but not by ketoprofen

**Fig. 2:**
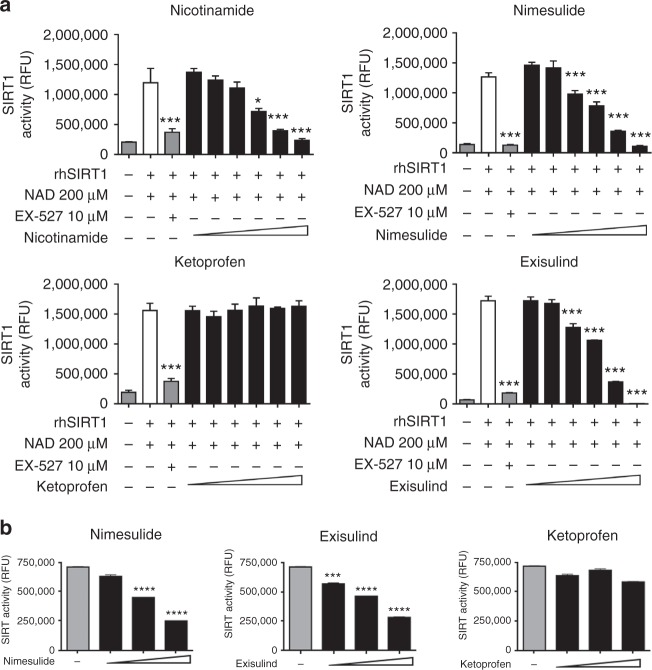
Nimesulide, diclofenac and exisulind, but not ketoprofen, inhibit rhSIRT1 activity in vitro. Data from more NSAIDs are shown in Fig. S2B. **a** rhSIRT1 activity was measured in vitro with a fluorescence-based assay in the presence of 200 μM β-nicotinamide adenosine dinucleotide (NAD) and of increasing concentrations (11, 33, 100, 300, 900, 2700 μM) of nicotinamide and of the indicated NSAIDs; treatment with 10 μM EX-527, a selective inhibitor of rhSIRT1, was used as positive control of the reactions. Data are represented as mean ± SEM. *P < 0.05 **P < 0.01 ***P < 0.001 *versus* the value of the NAD treated sample; *P*-values were calculated by two-way ANOVA followed by Bonferroni’s test. **b** The sirtuin activity was tested in MDA-MB-231 protein extracts treated with increasing concentrations (300, 900, 2,700 μM) nimesulide, ketoprofen and exisulind. Data are represented as mean ± SEM. *P < 0.05 **P < 0.01 ***P < 0.001 *versus* the value of the NAD treated sample; *P*-values were calculated by two-way ANOVA followed by Bonferroni’s test from endogenous SIRT activity

**Fig. 3:**
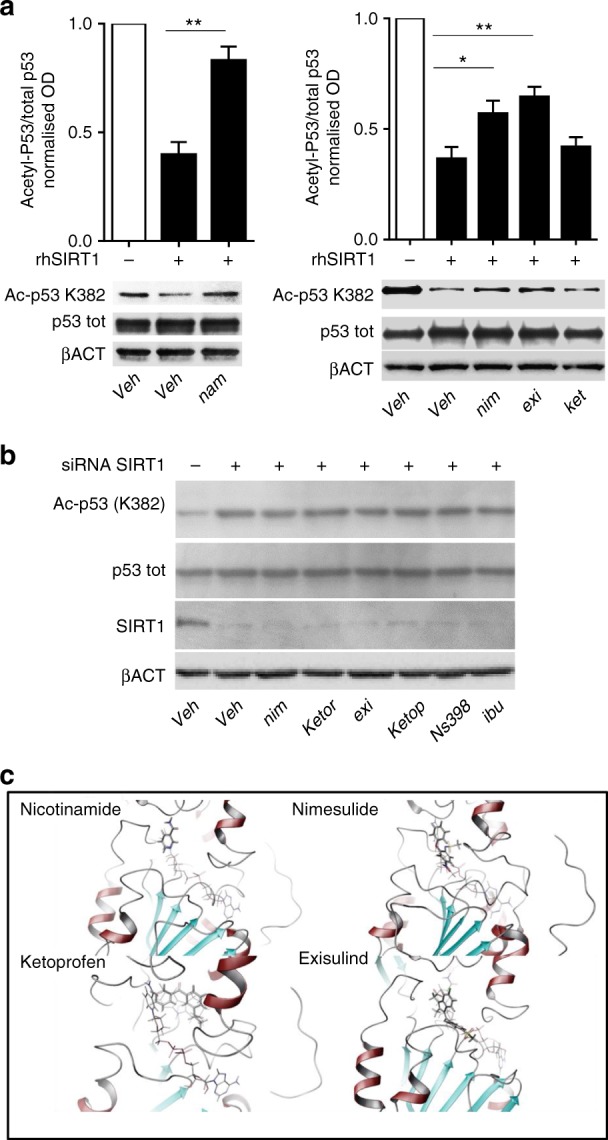
The NSAIDs-induced acetylation at the K382 residue is mediated by SIRT1 inhibition. **a** Deacetylation assay on native p53 protein. Picture shows a representative immunoblot analysis of native acetylated and total p53 present in the extracts of MDA-MB-231 6 h before harvesting, cells were treated with 20 etoposide μM to obtain sufficient amount of p53 acetylation; the same batch of protein extract was divided in 15 µg aliquots and treated either with vehicle (*veh*) or with 300 µM nicotinamide (*nam*), nimesulide (*nim*), exisulind (*exi*), ketoprofen (*ket*) as described in Materials and Methods paragraph, the extract was then treated with rhSIRT1 or with saline buffer. β-actin is reported as loading control. Quantification of the immunoblot signals are reported in the inserted graph: acetylated p53 signal was normalised on the corresponding total p53 signal and referred to the veh/- SIRT1 sample. Bars represent the average ± SEM normalise values of three independent experiments. *P *<* 0.05 **P < 0.01 *P*-values were calculated by Student’s *t*-test. **b** Immunoblot analysis of p53 acetylation at K382 site in SIRT1 knock-down MDA-MB-231 cells. MDA-MB-231 expressing siRNA SIRT1 were treated with 270 μM vehicle, nimesulide, ketorolac, exisulind, ketoprofen, NS398, ibuprofen. β-actin is reported as loading control. **c** Best docking poses for selected SIRT-1 inhibitors. Data from more NSAIDs are shown in Fig. S3A. The enzyme is shown as ribbon, inhibitors are shown in stick representation. Nicotinamide, nimesulide, and exisulind overlap NAD binding site; ketoprofen overlaps the EX527-analog binding site only after its relevant structural deformation

The activity of exisulind and the fast dynamics of p53 acetylation observed led us to hypothesise a COX-independent mechanism modulating the activity of enzymes able to deacetylate or acetylate the K382 residue; thus, we tested the effects of NSAIDs on the two enzymes known to add (P300) or remove (SIRT1) the acetyl residue at the K382 site of p53. The study was done in vitro by using recombinant human P300 (rhP300) acetylase and the recombinant human SIRT1 deacetylase (rhSIRT1).^27,28^ The assay for measuring P300 activity was based on the ability of the enzyme to transfer the radioactive ^3^H-acetyl from ^3^H-acetil-CoA to a substrate histonic peptide (for the P300 assay), whereas for SIRT1, we measured the fluorescence produced by a two-step reaction initiated by the acetyl removal from a fluorescence-substrate peptide containing an acetylated lysine followed by the cleavage of the substrate and the release of a highly fluorescent residue. While no effect on rhP300 activity was observed (Figure S2A), all NSAIDs tested and exisulind, but not ketoprofen inhibited the rhSIRT1 activity in a concentration-dependent manner (Fig. 2a and Figure S2B); for most of the compounds, the inhibition was in the same order of potency of the physiological inhibitor nicotinamide, suggesting the likelihood of a similar effect in vivo (Fig. 2a, Figure S2B and C, Table S1); in agreement with this conclusion, the nicotinamide treatment of MDA-MB-231 cells increased the p53 acetylation at similar concentrations of NSAIDs (Figure S1A). Due to the limitation of the SIRT-1 fluorescence-based assay,^29,30^ we further tested the inhibitory action of nimesulide on rhSIRT1 using a bioluminescent assay^31^: the results obtained were superimposable (Figure S2C). The inhibitory action of NSAIDs and exisulind was also observed using the endogenous SIRT1, as demonstrated by performing the fluorescent assay with the MDA-MB-231 protein extracts (Fig. 2b). Moreover, as an additional demonstration of the direct effect of SIRT1 on the K382 residue of p53, we investigated the deacetylation potential of rhSIRT1 on the acetylated p53 present in MDA-MB-231 cells. The presence of rhSIRT1 alone decreased by 64% the content of acetylated p53, but the activity of the enzyme was significantly inhibited by adding 300 µM nicotinamide (Fig. 3a); with this assay the same concentration of nimesulide and exisulind, but not ketoprofen were also able to inhibit the enzyme (Fig. 3a), thus supporting the notion that these effects were directly mediated by SIRT1. To further strengthen this conclusion, we knocked down SIRT1 in MDA-MB-231 cells by generating stably transfected clones with an expression vector encoding for a siRNA directed against SIRT1 mRNA.^19^ The effective knock down of SIRT1 expression was demonstrated by western blot analysis, whereas, we also detected a constitutive increment of the p53 basal acetylation at the K382 residue. After treatments with NSAIDs and exisulind (Fig. 3b), the basal acetylation remained unchanged firmly demonstrating the direct role of SIRT1 in mediating the K382 acetylation induced by these compounds in breast cancer cells.

Next, we asked whether the SIRT1 inhibitory action of the NSAIDs and exisulind was due to a direct interaction with the deacetylase, and what the molecular basis of this inhibition was. To this aim, we have carried out an accurate molecular docking procedure both on its *holo* and its *apo* structure^32^ obtained by removing in silico the NAD cofactor and the EX-527 inhibitor. The docking data showed that all the tested compounds were able to bind the inhibitor pocket of SIRT1 (Fig. 3c and Figure S3). The mechanism of action proposed for EX-527 was linked to its ability to induce an extended NAD conformation thus blocking the access to the channel of the acetylated lysine substrate.^33^ Some of the NSAIDs (e.g., ibuprofen, diclofenac) were able to bind at the same position of EX-527,^32,33^ and for them, we hypothesised a similar NAD-dependent inhibitory mechanism. Differently, compounds containing condensed heteroaromatic rings, such as indomethacin and its derivatives, generated steric hindrance also with the extended NAD conformation, suggesting they can bind SIRT1 only through a direct competition with NAD. This differential behaviour could be argued also from a comparative analysis of the docking scores of all the tested compounds obtained with and without NAD (Table 1). These scores are suitable for classification purposes and useful for deciphering the molecular mechanism of the investigated ligands, but they are not directly related to ligand affinity. Compounds preferentially binding the *apo* protein, partially overlapping the NAD binding site, can be classified as competitive ligands with respect to NAD; whereas compounds with the most favourable docking scores on the *holo* protein, and occupying the EX-527 binding site, can be classified as mixed inhibitors, able to both induce a NAD distortion misconformation and its displacement (Fig. 3c and Figure S3). Our proposed classification was further confirmed by performing the same docking analysis and energy evaluations on a more recent X-ray *apo*-SIRT1 structure crystallised in the presence of an active-site directed inhibitor that occupies the peptide and NAD^+^-binding sites.^34,35^ As expected by a mixed inhibitory effect, the calculated affinity (Table 1) did not correlate with the enzymatic inhibition (Fig. 3c and Figure S2). In the extreme case of ketoprofen, which did not show any inhibitory activity in reference assays, we carried out a more in depth molecular docking search (Glide XP). From this further analysis, we identified the peculiar rigidity of ketoprofen structure induced by an extended mesomery as the main reason for its lacking inhibitory activity. In fact, ketoprofen has a very low affinity for the EX-527 binding site, since it engages A262 and F297 in steric clashes (Figure S3B), producing the lowest binding free energy/affinity (MM-GBSA) among all the tested compounds for SIRT1 (Table 1). Thus, the docking data confirmed the ability of NSAIDs to directly interact with the NAD cleft of SIRT1 and to inhibit the deacetylase activity through competitive or non-competitive mechanisms depending on their binding fashions.

**Table 1:** Molecular docking data and affinity values (binding free energies)

	*Apo* 4I5I	*Holo* 4I5I	
Molecules	Docking score [kcal/mol]	Docking score [kcal/mol]	MM-GBSA [kcal/mol]
sulindac sulfide	−6.8	–	−30.3
NS-398	−5.7	−6.5	−43.8
nimesulide	−6.4	−6.0	−46.0
ketoprofen^a^	−5.2	−3.3	−24.7
EX527	–	−9.2	−67.5
ibuprofen	−6.5	−7.2	−42.1
ketorolac	−7.2	−7.6	−36.2
sulindac	−6.5	–	−30.3
exisulind	−7.0	–	−29.7
diclofenac	−6.9	−7.4	−31.1
nicotinamide^a^	−5.8	–	−33.7
NAD	−16.5	–	−79.5

^a^Poses generated by Schrödinger Glide XP

### NSAIDs and exisulind activates p53-mediated transcription in vivo

**Fig. 4:**
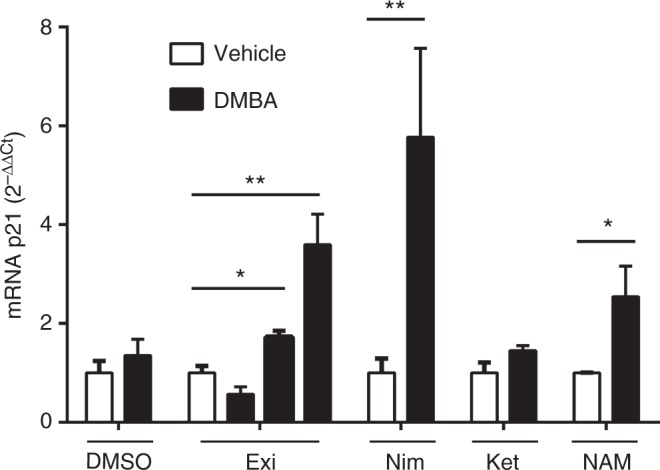
NSAIDs exisulind and nicotinamide treatment, but not ketoprofen increases p53 activity in vivo. Five female mice per group were treated *per os* (gavage) with a daily dose of 3.75, 7.5, 15 mg/Kg exisulind (exi), 15 mg/Kg nimesulide (nim), 15 mg/Kg ketoprofen (ket), 15 mg/Kg nicotinamide (NAM) or dimethyl sulfoxide (DMSO, vehicle). Treatment was carried out for eight days; at day 5 a single dose of an acetone solution of 12 mM DMBA (left mammary gland) or acetone (right mammary gland) was injected in the mammary fat pad of the animals (Figure S4B). p21 mRNA expression was determined by real-time PCR; bars in the graph are the average ± SEM values quantified with the 2^−∆∆Ct^ method. * *P* < 0.05; ** *P* < 0.01 DMBA *versus* acetone treated breast. *P-*values were calculated by Student’s *t*-test

Activation of the p53 pathway through SIRT1 inhibition has been shown to have powerful anticancer effects^36–38^ and oral nicotinamide, the natural SIRT1 inhibitor, was shown to reduce the rate of non-melanoma skin cancer in a phase III clinical trial.^39^ The increased K382 acetylation of nuclear p53 (Figure S1B), suggested that the oncosuppressor is selectively induced by NSAIDs, therefore, we investigated whether these drugs were able to increase the level of p21 expression, an anti-proliferative gene directly modulated by the p53 transcriptional activity.^40^ The treatment with nimesulide and exisulind of two immortalised cell lines carrying wild type p53 (human breast epithelial hTERT-HME1 and mouse myoblast C2C12 cells) was able to increase the expression of the p53 target gene p21, suggesting that the oncosuppressor is transcriptionally activated by these drugs (Figure S4A). Based on these findings, we investigated the effect of the anti-inflammatory drugs in an animal model of the initial transformation stage, e.g., local treatment of the mammary gland with the carcinogen 7,12-dimethylbenz(a)anthracene (DMBA). As illustrated in the Figure S4B, mice were treated for 8 days with 15 mg/Kg/day nimesulide, 15 mg/kg/day ketoprofen, 3.75-7.5-15 mg/Kg/day exisulind, 15 mg/Kg/day nicotinamide or vehicle *per os*. At day 5 from the beginning of the treatment, the left mammary gland was injected intra fat pad with DMBA and the right mammary gland was treated with vehicle (acetone). In the left mammary gland, p21 mRNA expression was clearly induced in the mice treated with nimesulide, exisulind and nicotinamide (Fig. 4); such an increase was not observed in mice treated with ketoprofen. These experiments confirmed in an in vivo model that NSAIDs (but not ketoprofen), exisulind and nicotinamide were able to increase p53 activity; interestingly, the exisulind active dose (15 mg/Kg) was of the same order of magnitude of the dose reported to obtain polyp regression in humans.^15,16^ To verify that the NSAID-mediated p53 activation was specifically occurring in the tissue exposed to genotoxic stress and not in others, we repeated the experiment (Figure S4B) with the p53 reporter mouse.^41^ The reporter mouse was generated with a knock-in strategy described by Tinkum and colleagues^42^ (Figure S4C) obtaining a model where the generalised expression of the luciferase reporter is directly proportional to the p53 state of transcriptional activation and can be measured in vivo by bioluminescence-based imaging (BLI).^22^ In this model, the bioluminescence in the mammary glands exposed to DMBA was highest in the mice treated with nimesulide (15/mg/Kg/day *per os*) (Figure S4C and D) and no increased bioluminescence was detectable in body areas other than the DMBA-treated breast. This experiment suggested that the NSAID-mediated activation of p53 was restricted to the cells proliferating after the genotoxic stimulus and not to other physiologically proliferating tissues in the mouse body (i.e. bone marrow in the legs and thorax areas).

### SIRT1 inhibition counteracts tissue proliferation produced by DMBA

**Fig. 5:**
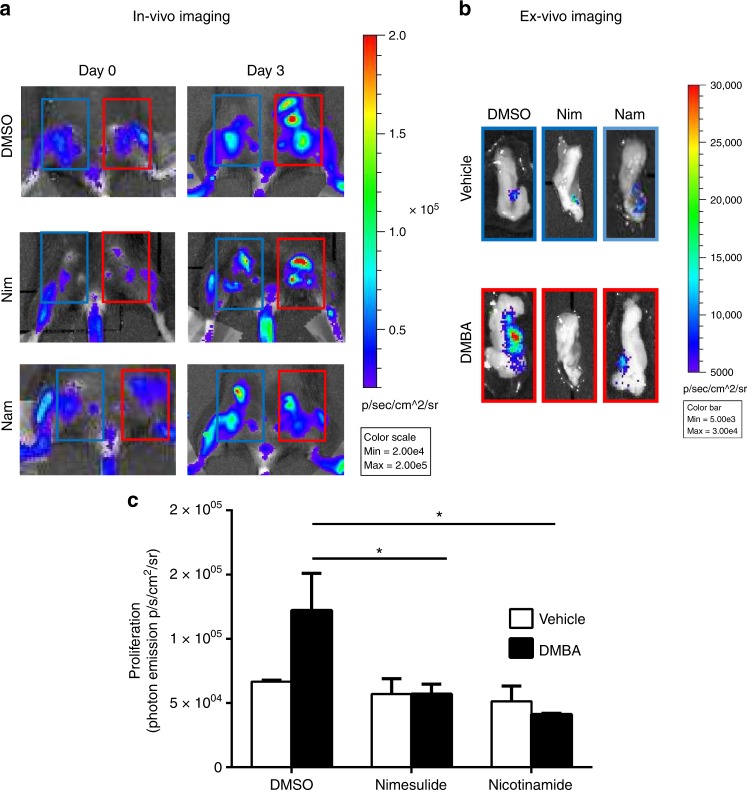
NSAIDs, exisulind and nicotinamide treatments, but not ketoprofen decrease tissue proliferation in vivo. Five females repTOP*mito*IRE mice/group were treated with nimesulide (Nim), nicotinamide (Nam) or vehicle (DMSO) following an identical protocol described in Fig. 4 and Figure S4B. Pictures show the bioluminescence emission marking the proliferative activity in the repTOP*mito*IRE reporter mouse (**a**) or in the dissected mammary glands (**b**). Data from more NSAIDs are shown in Fig. S5A. **c** Quantification of the photon emission as a measure of tissue proliferation from the dissected mammary glands; bars are the average ± SEM values of the photon emission normalised over the area of the acquisition surface (p/s/cm^2^/sr). *P < 0.05 mammary glands treated with DMBA and 15 mg/Kg nimesulide (Nim) or nicotinamide (Nam) versus DMSO treated animals; *P-*values were calculated by Student’s *t*-test

Next, we investigated whether the increased p53 activity in the DMBA treated mammary glands correlated with a decreased cell proliferation. To this aim, we carried out the DMBA treatment as before (Figure S4B) in another model of luciferase reporter mouse enabling to measure by BLI cell proliferation (the repTOP*mito*IRE).^21^ As observed in the p53 reporter mouse, the effect of NSAIDs was selectively detected in the genotoxic treated tissue, no effect was observed in other physiologically proliferating tissues i.e., bone marrow (Fig. 5). Ex vivo analysis of the photon emission in the mammary glands treated with DMBA demonstrated that cell proliferation was significantly lower in the animals treated with NSAIDs, exisulind and nicotinamide (Fig. 5 and Figure S5A); as also previously demonstrated,^21^ in repTOP*mito*IRE reporter mice, reduction of the bioluminescent signal correlated with a decreased Ki-67 immunostaining (Figure S5B). This experiment indicated that treatment with NSAIDs, exisulind and nicotinamide attenuated tissue proliferation induced by the exposition to a genotoxic agent, a mitogenic signal which is known to be responsible of the early clonal expansion of mutagenised cells. Once more, ketoprofen did not have the same effects of NSAIDs.

### Intraoperative ketorolac treatment induces p53 acetylation at the K382 site

**Fig. 6:**
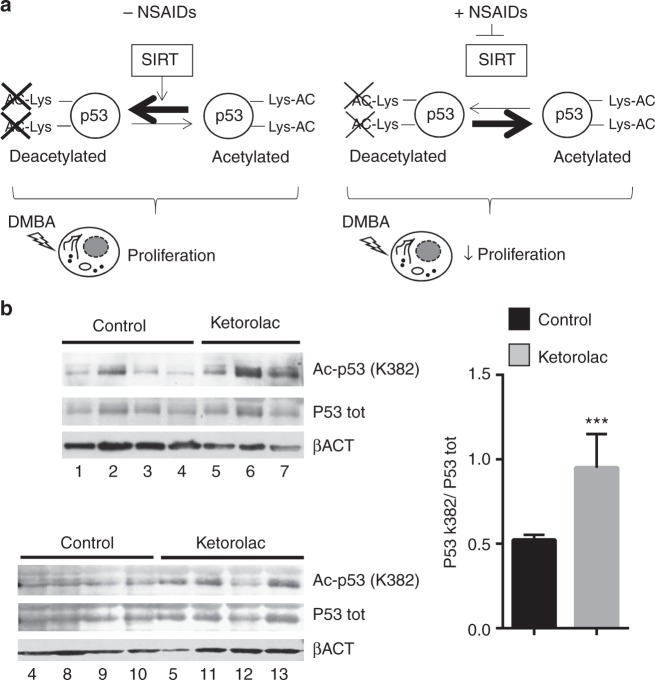
Intraoperative ketorolac induces p53 acetylation at the K382 site in clinical settings. **a** Model of the NSAIDs and exisulind chemopreventive action: the direct inhibition of SIRT1 leads to the unbalance activity of p53 involved in the early steps of tumourigenesis. A genotoxic stress increases proliferation (left panel, − NSAIDs) which is counteracted by NSAIDs (right panel, + NSAIDs). **b** Immunoblot analysis was carried out using anti-acetyl (K382) p53 and anti-total p53 antibodies and protein extracts obtained from 13 patients: six were treated with ketoroloac (20 mg *per os*) 2 h before surgery and seven with opiates for intraoperative analgesia. β-actin is reported as loading control. Bars in the graphs represents densitometry quantifications of the autoradiographic signals (acetylated p53 vs total p53); ***P < 0.001 versus the level of control; *P-*values were calculated by Student’s *t*-test

In a previous epidemiological study, Forget and collaborators suggested that an intraoperative treatment with the NSAID ketorolac was associated with a reduced relapse likelihood in the first 24 months, improved disease-free survival and overall survival in patients undergoing mastectomy;^8^ interestingly, these beneficial effects were particularly relevant for high-body mass index group of patients.^12^ Moreover, a recent report demonstrated the positive effects on survival of the intraoperative ketorolac also in the treatment in ovarian cancer;^9^ interventional trials are underway to test the beneficial effects of this procedure. To investigate the potential clinical relevance of the mechanism described in our study, we verified whether this treatment could induce p53 acetylation at K382 site. Immunoblot analysis showed a significant increase of K382 acetylation in the tumours obtained from patients (Table S2) treated with ketorolac as compared to controls (treated with opiates), indicating that SIRT1 inhibition could be promoted by a single NSAID treatment during surgery (Fig. 6b).

Altogether, our data suggest a therapeutic strategy for chemoprevention based on the direct inhibition of SIRT1 deacetylase by NSAIDs-like molecules; this inhibitory effect is likely to modulate the activity of specific SIRT1 targets^43^ responsible for the early tumourigenesis steps promoted by the exposition to genotoxic agents (Fig. 6a). In particular, our study links together the NSAIDs chemopreventive activity with the well-known SIRT1/p53/P21 anti-oncogenic pathway, suggesting a novel strategy for the design of tumour protective drugs.

## Discussion

Our work identifies for the first time SIRT1 as a direct target of NSAIDs and demonstrates that this interaction underlies the anti-proliferative effects of these drugs through a COX-independent mechanism. We show that the deacetylase inhibition triggers an oncosuppressor signal able to prevent cellular functions important for the initial stage of the neoplastic transformation. Our findings designate a novel therapeutic strategy for cancer chemoprevention. Indeed, NSAIDs-like drugs can be ideal prototype, as they were shown to decrease the risk of several cancers in epidemiological studies and to prevent neoplastic transformation when administered to humans.^9,12,15^ Since most of the toxicities manifested by these drugs in chronic treatments can be ascribed to COX inhibition, our data by distinguishing the anti-inflammatory and chemopreventive actions, as due to the inhibition of two distinct targets, provides the rationale for the design of safer NSAIDs without COX inhibitory activity, but retaining the anti-cancer, beneficial effects obtained with SIRT1 inhibition. The hypothesised therapeutic strategy is feasible as the IC50s calculated in vitro for NSAIDs (Table S1) are in the order of magnitude of the natural SIRT1 inhibitor nicotinamide, currently used in long term treatments,^39^ and the active dose in vivo (Fig. 2 and Fig. 4) is comparable with the dose efficiently used to treat patients (Fig. 6b).^15,16^ It is true that the IC50s calculated for NSAIDs, exisulind and nicotinamide are much lower compared to EX-527 or other powerful SIRT1 inhibitors. However, nicotinamide that has been investigated in a phase 3 clinical trial for skin-cancer prevention (our data provide a strong support to evaluate this preventive treatment for other cancer types) and exisulind itself have already demonstrated efficacy in clinical trials: these are direct convincing evidences in favour of the development of weak SIRT1 inhibitors. The development of NSAIDs-derived SIRT1 inhibitors may provide a valid alternative in case the chemopreventive activity of nicotinamide would not be proven efficacious for tumours other than skin-cancers. Future studies should clarify for chronic treatment of patients at risk of cancer, whether a less tight SIRT1 inhibition is indeed sufficient for cancer prevention with fewer side effects, compared to high-affinity SIRT1 inhibitors under development as anti-cancer drugs.

SIRT1 as a therapeutic target in tumourigenesis has been ground for debates on two conflicting, dichotomic thesis, which postulated its deacetylase enzymatic activity as pro- or anti-oncogenic.^44^ The link of NSAIDs with the SIRT1/p53 pathway was raised also by other groups reporting the pro-senescence effects of aspirin in breast^25^ and colon cancer cell lines,^45^ although a direct effect of aspirin on SIRT1 activity has never been demonstrated. In keeping with our study, Alfonso and collaborators showed that aspirin induces p53 acetylation and a pro-apoptotic effect in breast cancer cell line, while the study from Jung and collaborators proposed SIRT1 as a key mediator for the pro-senescence activity of aspirin in colon cancer. The latter study reported a biphasic effect of aspirin on SIRT1 activity with an initial activation, followed by inhibition of the deacetylase at later time points. The early increase of SIRT1 activity observed by this group might be a consequence of the peculiar metabolic effects of aspirin, which transiently increases the cellular levels of cAMP and NAD^+^, the physiological activator of SIRT1.^45^

Other studies have pointed out the effects of SIRT1 inhibition and p53 acetylation in colon cancer cell lines with controversial results even in the same cell lines.^45–50^ Nevertheless, most of the reports are in line with an apoptotic or anti-proliferative effect of SIRT1 inhibition and p53 acetylation also in many breast cancer cell types.^36,51–57^ These controversial data might be ascribed either to off target activities of different SIRT1 inhibitors used in these studies or to different culture passages of the same cell line that might have acquired additional genetic properties in culture, which changed the cellular responses to the modulation of the SIRT1/p53 signalling. Thus, in our study we have chosen to demonstrate in cell lines the existence of the novel mechanism shared by exisulind and those NSAIDs that displayed in previous studies anti-cancer properties, but not ketoprofen for which an anti-cancer activity was never reported. To avoid the variability intrinsic in cell lines, we decided to test the effect of NSAIDs on tissue proliferation in vivo in a mouse model of early mammary gland transformation, where we demonstrated that the inhibition of SIRT1 is perfectly correlating with the in vivo ability of these compounds to activate p53 and preventing tissue proliferation during the early transformation steps. Again, ketoprofen was not able to elicit any in vivo effect on tissue proliferation. Collectively our in vitro, cell culture and in vivo data demonstrated that SIRT1 inhibition produces anti-proliferative effects on DMBA damaged tissues; thus, at least in the initiation phase of neoplastic transformation, SIRT1 inhibition and the subsequent activation of the deacetylase target genes seem to be positive events producing a cell cycle arrest of mutated clones. In addition to the anti-proliferative effects we have characterised, it should also be mentioned that p53 signalling may not be the only pathway involved in the chemopreventive activity of NSAIDs, since SIRT1 is able to modulate a variety of key targets involved in the control of apoptosis, DNA repair, inflammation, energy metabolism, angiogenesis and cell proliferation, signals that could play a significant role in the anti-cancer properties of NSAIDs.^58,59^ Moreover, the increased p21 expression observed upon NSAIDs treatment might also be promoted by pathways independent from p53.^60^ Future studies should address the extent to which these other signalling pathways contribute to the anti-cancer activity.

The ability of ketorolac to increase K382 acetylation in mastectomy patients directly links SIRT1 inhibition with the decreased relapse and increased survival associated with the treatment.^8,12^ Thus, our results provide the rational basis for a prospective clinical trial aimed at demonstrating the beneficial effect of intraoperative ketorolac as well as to assess the effect of the chronic nicotinamide administration to mastectomy patients. Both are non-toxic and cheap treatments eliciting SIRT1 inhibition with the potential of significantly improving the management of breast cancer patients.

In conclusion, the demonstration of a new target for NSAIDs responsible of their chemopreventive activity provides a novel solution for an important medical need. In the last decades, the system biology has made great progress in the discovery of prognostic markers characterizing populations at higher risk for several types of cancer; it is now compulsory to move forward from prototype molecules to the development of preventive drugs for the cancer therapeutic area and to provide clinicians with a correct armamentarium needed for treating subjects at high risk of cancer outgrowth.

## Supplementary information

Supplemental Figure, table, material and methods
