# Supplementary material for: Inhibition of SIRT1 deacetylase and p53 activation uncouples the anti-inflammatory and chemopreventive actions of NSAIDs
Source: Br J Cancer. 2019 Feb 11;120(5):537–46. doi: 10.1038/s41416-018-0372-7 (PMC6461760; doi:10.1038/s41416-018-0372-7)
Supplement: Supplementary file 1 — Supplemental Figure, table, material and methods [file 41416_2018_372_MOESM1_ESM.docx]

**SUPPLEMENTARY FILES**

**Supplementary Materials and Methods**

**Figure Supplements**

Figure S1. Treatment with ibuprofen, NS398, sulindac sulfide, ketorolac, nicotinamide increase p53 acetylation at K382 sites

Figure S2. NSAIDs inhibit SIRT1 deacetylase activity while it does not influence rhP300 acetylase activity

Figure S3. Best docking poses for selected SIRT-1 inhibitors

Figure S4. NSAIDs induces the expression of P53 target gene p21

Figure S5. Effects of exisulind and ketoprofen on tissue proliferation

**Table supplements**

Table S1. IC50 calculated from the in vitro assay

Table S2. Patients: diagnosis, treatments and tumor features

**Materials and Methods**

**Cell culture**

C2C12 (mouse myoblast) and HTERT-HME1 (human mammary epithelium) cell lines were purchased from the American Type Culture Collection (ATCC, Manassas, VA). C2C12 were grown in RPMI 1640 medium (Life Technology) supplemented with 10% FBS (Sigma-Aldrich) and streptomycin-penicillin (50.000 IU plus 50 mg per liter). HTERT-HME1 were grown in Dulbecco’s Modified Eagle’s Medium (DMEM, Life Technology) supplemented with 10% FBS (Sigma-Aldrich), streptomycin-penicillin (50,000 IU plus 50 mg per liter), 100 ng/µl Endothelial Growth Factor (EGF, Sigma-Aldrich) and 0,5 ng/ µl hydrocortisone (Sigma-Aldrich).

**SIRT1 activity assays**

Abcam’s SIRT1 Activity Assay Kit (Fluorometric ab156065) was used to quantify SIRT1 activity according to the manufacturer’s protocol. In addition, SIRT1 activity was also measured with a bioluminescent assay^29^ (SIRT1-GLO assay, Promega) according to the manufacturer’s instructions. Briefly, rhSIRT1 was incubated in the presence of NAD and of an acetylated SIRT1 substrate peptide covalently linked to luciferin (GLO). In the reaction, SIRT1 deacetylates the substrate peptide unmasking a site for a subsequent peptidase reaction, which can thus release free luciferin from the substrate: the amount of luciferin in the mixture is proportional to the deacetylase enzymatic activity and can be quantified by measuring the photon emission upon addition of the luciferase enzyme to the reaction mixture.

Quantitative measurement of SIRT1 activity in 5 µg of MDA-MB-231 cell lysate cell lysates was carried out with a commercial kit (ab156065, Abcam) according to the manufacturer’s protocol.

**Real Time PCR**

To amplify the human 36B4 and p21 genes were used the following primers: 36b4 forward 5’- ggcgacctggaagtccaact-3’, reverse 5’-ccatcagcaccacagccttc-3’; P21 forward 5'- tggagactctcagggtcgaaa-3', reverse 5’-gcgtttggagtggtagaaatctg-3’. Data were analyzed using the ABI Prism 7000 SDS Software and 2^-ΔΔCt^ method. The levels of genes mRNA transcripts were normalized on the constitutively expressed gene 36b4.

**Immunohistochemistry.**

Mammary fat pads were fixed in 10% neutral buffered formalin, routinely processed and embedded in paraffin blocks. Four µm thick sections obtained from these blocks were then immunostained with a primary rabbit monoclonal antibody against Ki-67 antigen (#RM-9106-S, LabVision) using a standard immunoperoxidase protocol (BA-1000 Biotinylated Goat Anti-Rabbit IgG Antibody and PK-6100 Vectastain Elite ABC kit, Vector Laboratories) followed by diaminobenzidine chromogen reaction (SK-4100 Peroxidase substrate kit DAB, Vector Laboratories). Serial sections incubated with normal goat serum instead of the primary antibody served as negative control.

**Statistical analysis and IC_50_ and K_i_ determination.**

Data analyses were performed using GraphPad 5 Instat software® (GraphPad Prism Inc. San Diego, CA, USA), we have applied Bonferroni’s and Student’s *t* test analysis for determining statistical significance. IC_50_ was measured, according to GraphPad 5 Curve Fitting Guide (GraphPad Software), with log(inhibitor) *versus* response–Variable slope equation. Ki of tested compounds was calculated using Ki= IC50/(1+[Substrate]/Km) equation where K_m_ for the rhSIRT1 for the fluoro-substrate peptide (final concentration 20µM) was assumed = 4 as reported in the CycLex SIRT1/Sir2 Deacetylase Fluorometric protocol (Cyclex Catalogue CY-1151V2).

**Nuclear and cytoplasmic protein extracts**

MDA-MB-231 cells treated with NSAIDs were collected in PBS buffer supplemented with protease inhibitor cocktail (Roche). After 4 min centrifugation at 2000 x rcf, cell pellets was suspended in lysis buffer (50 mM Hepes-KOH, pH 7.5; 140 mM NaCl; 1mM EDTA; 10% Glycerol; 0.5% NP-40; 0.25% Triton X-100 and protease inhibitor cocktail) rocking at 4°C for 10 min and checked at the microscope for cytoplasm removal and the presence of intact nuclei. Nuclei preparation was then spin at 2,000 × rcf for 4 min at 4°C. Supernatant was collected as cytoplasmic protein fraction while nuclear fraction was obtained by suspending pellets of nuclei in lysis buffer (10 mM Tris-Hcl, pH 7.4, 450 mM NaCl, 15% glycerol, 1% Triton-X-100, 1 mM sodium orthovanadate, 10 μg/ml leupeptin, 10 μg/ml aprotinin, 1 mM NaF, protease inhibitor cocktail and 1 mM PMSF); nuclear membrane was disrupted by three cycles of freezing and thawing and the supernatant was collected after 30 min minifuge centrifugation at the 13000 x rcf.


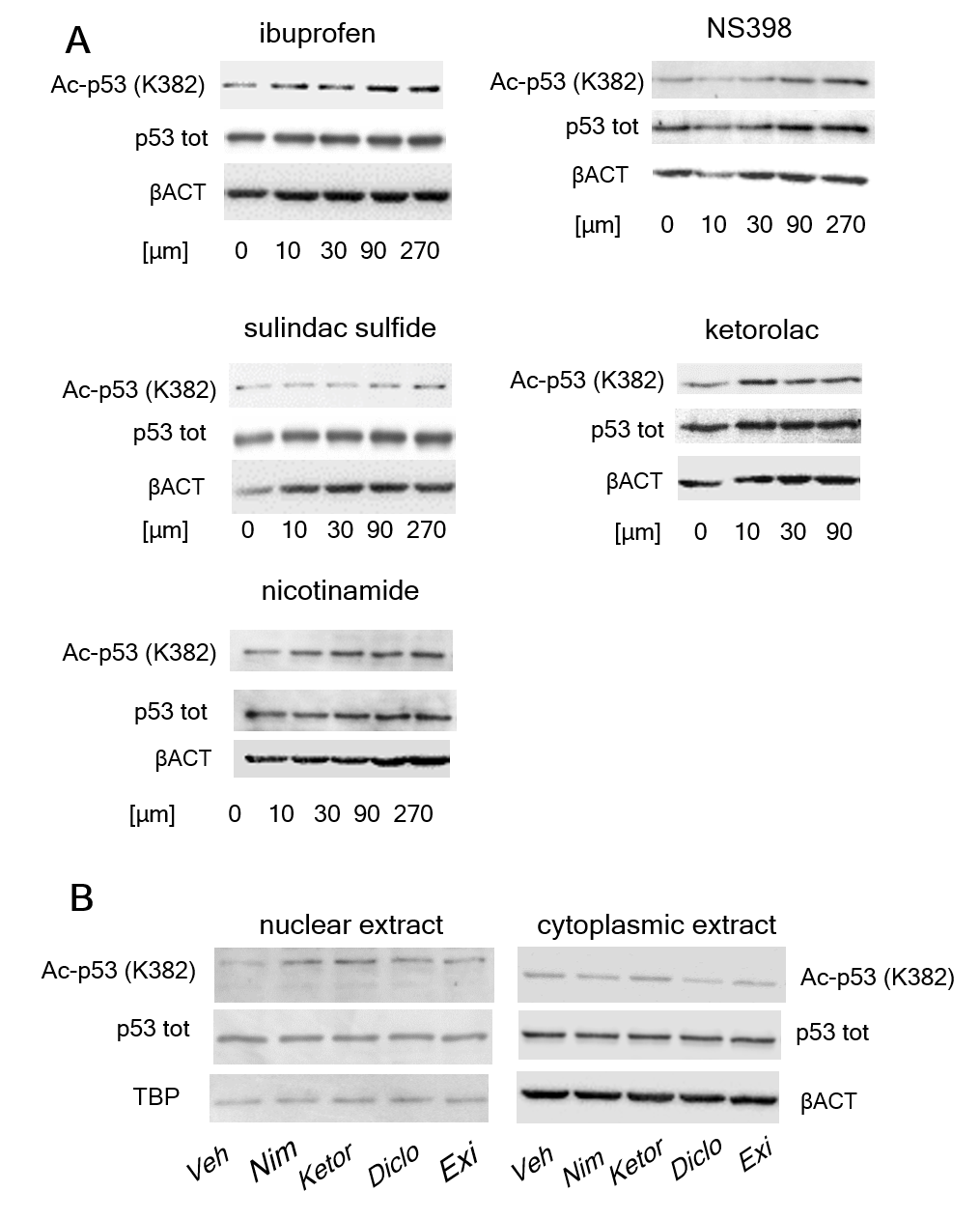


**Figure S1. p53-K382 acetylation in MDA-MB-231.** (**A**) Treatment with ibuprofen, NS398, sulindac sulfide, ketorolac and nicotinamide increases p53 acetylation at the K382 site; immunoblot analysis was carried out using anti-acetyl (K382) p53 and anti-total p53 antibodies and protein extracts obtained from MDA-MB-231 cells treated for 3 hours with increasing concentrations of the indicated NSAIDs and of nicotinamide, β-actin is reported as loading control. (B) NSAIDs and exisulind increase the levels of acetylated p53 at the K382 residue in the nucleus but not in the cytoplasm. Veh=vehicle, Nim= nimesulide, Ketor= Ketorolac, Diclo= Diclofenac, Exi= Exisulind. TATA binding protein (TBP) is reported as nuclear loading control and β-actin is reported as cytoplasmic loading control.

**
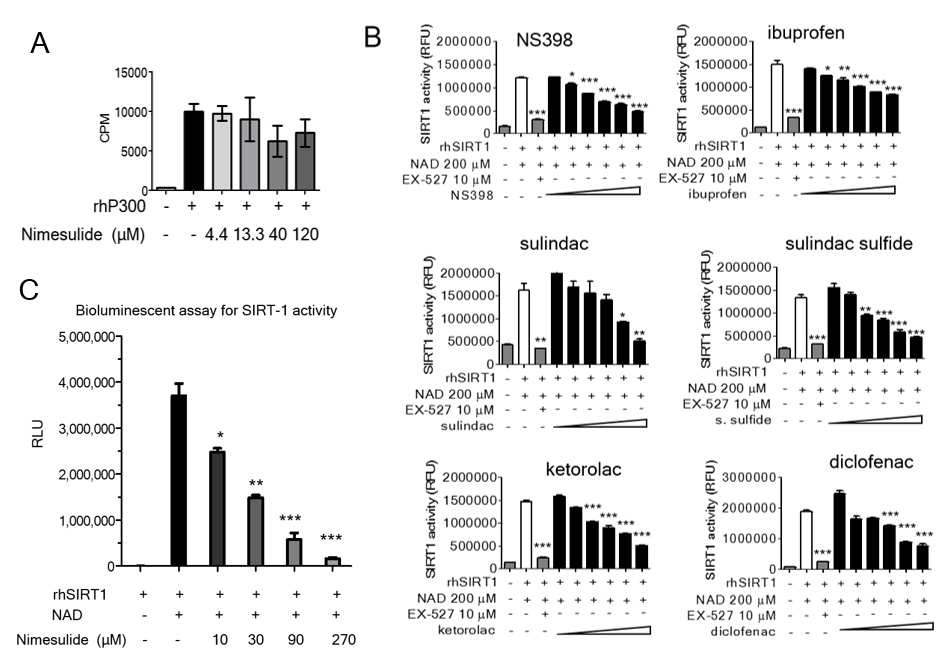
**

**Figure S2. NSAIDs inhibit SIRT1 deacetylase activity.** (**A**) The enzymatic activity of rhP300 was measured in the presence of increasing concentration (4.4-13.3-40-120 μM) nimesulide by using a commercial kit (Perkin-Elmer Life Sciences) based on the transfer of a radioactive ^3^H-acetyl from ^3^H-acetil-CoA to an histonic peptide; after the transfer the peptides are separated by a paper chromatography according to the manufacturer instructions. The amount of radioactivity incorporated by the peptides was determined with a β-counter. The assay was carried out in duplicates and repeated twice. (**B**) The enzymatic activity of rhSIRT1 was measured with a fluorescent assay according to the manufacturer instruction; the assay was carried out in the presence of increasing concentrations of the indicated NSAIDs. Bars represent the average values of rhSIRT1 activity measured in three experiments in duplicates; ****P*<0.001, ***P*<0.01 rhSIRT1 activity in the presence of NAD *versus* rhSIRT1 activity in the presence of NAD and of increasing concentration of the indicated NSAIDs. Statistical analysis was done using two-way ANOVA followed by Bonferroni’s analysis. (**C**), The deacetylase activity of rhSIRT1 was evaluated after treatment with 200 μM NAD and in the presence of the indicated nimesulide concentrations with a bioluminescent assay^29^ described in the Supplementary Methods section. The amount of photon emission was quantified with a luminometer and expressed as relative light units (RLU). Bars represent average value of two experiments carried out in triplicates. ****P*<0.001, ***P*<0.01 * *P*<0.05 rhSIRT1 activity in the presence of NAD *versus* rhSIRT1 activity in the presence of NAD and of increasing concentrations of nimesulide. Statistical analysis was done using two-way ANOVA followed by Bonferroni’s analysis.

**
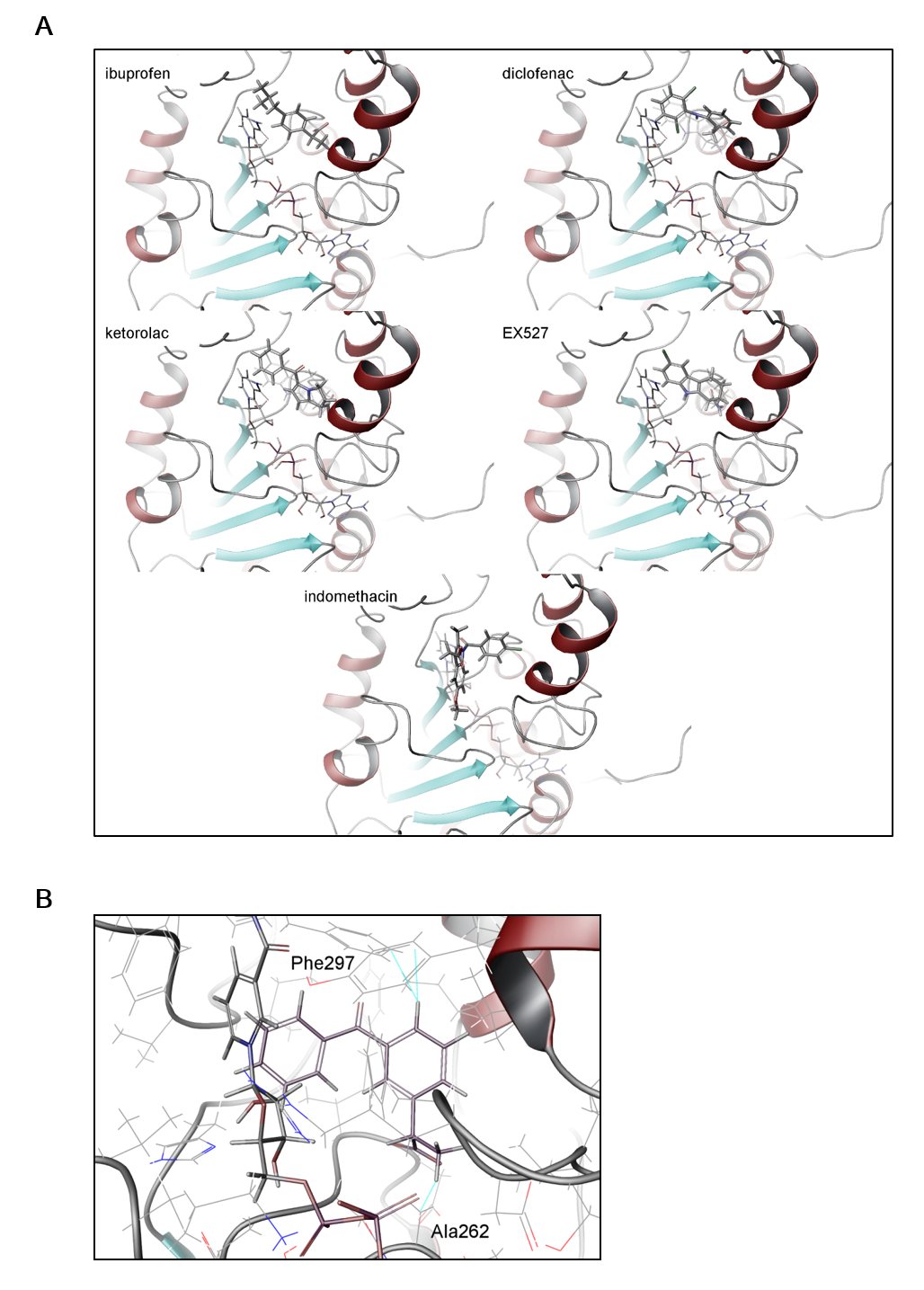
**

**Figure S3.** **Best docking poses for selected SIRT-1 inhibitors.** (**A**) Enzyme is shown as ribbon; inhibitors are shown in stick representation. Ibuprofen, diclofenac, ketorolac and EX527 overlap the EX-527 binding site; differently, indomethacin overlaps both the NAD and the EX527-analog binding sites. (**B**) Best docking pose for ketoprofen in the EX-527- binding site of SIRT1. Steric clashes with A262 and F297 are highlighted by dotted lines.

**
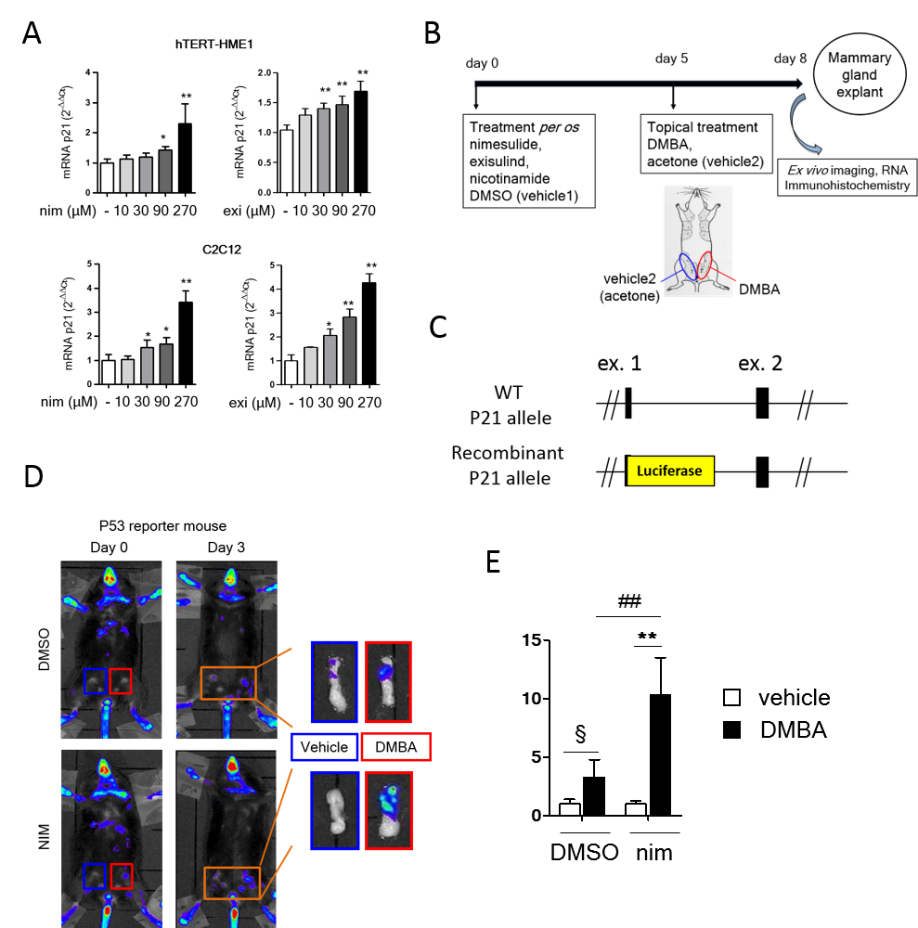
**

**Figure S4.** **NSAIDs induces the expression of P53 target gene p21**. (**A**) hTERT-HME1 and C2C12 are immortalized cells carrying wild type p53 and were treated with increasing concentrations of nimesulide (nim) and exisulind (exi) for 6 h. The expression of p21 was measured by real time PCR in both cell lines, bars represent average normalized values (quantified with the 2^-∆∆Ct^ method) of two independent experiments carried out in triplicates. ***P*<0.01, **P*<0.05.Statistical analysis was done using two-way ANOVA followed by Bonferroni’s analysis. (**B**) Experimental scheme to induce early tumorigenic events in the mammary glands of mice: five female mice/group were daily treated *per os* with exisulind, nimesulide, ketoprofen, nicotinamide or DMSO (vehicle1) for eight days. At day 5, a single dose of 50μl of an acetone solution of 12 mM DMBA (left mammary gland) or acetone (vehicle2, right mammary gland) was injected topically in the mammary fat pad of the animals. Imaging was carried out before DMBA injection (day 0) and before sacrifice (day 3); after sacrifice, mammary glands were collected for *ex vivo* imaging, gene expression and immunohistochemistry analysis. (**C**) The reporter mouse of p21 expression allows to measure p53 activity *in vivo*^41^ we generated and validated the p21 reporter mouse by knock in insertion of a luciferase reporter gene in frame with the endogenous mouse p21 gene, following the scheme previously reported by Tinkum and colleagues^42^. Two groups of five female p21 reporter mice were treated per os (gavage) with a daily dose of 15 mg/kg nimesulide or with DMSO (vehicle) for eight days; at day 5, a single dose of DMBA (left mammary gland) or acetone (right mammary gland) were injected in the mammary fat pad of the animals with the experimental scheme reported in (**B**). Representative pictures show the bioluminescence emission marking the p53 activity in the whole body of the p21 reporter mouse (left set of pictures) or in the dissected mammary glands (right set of pictures). Imaging was carried out before DMBA injection (day 0) and before sacrifice (day 3); after sacrifice, mammary glands were collected for *ex vivo* imaging and gene expression analysis. (**D**) Quantification of the p53 activity (photon emission) in the mammary glands by *ex vivo* imaging. Bars in the graphs are the average values of the photon emission normalized over the area of the acquisition surface (cts/s/cm^2^), ** P<0.01, § P<0.05, DMBA treated mammary glands versus acetone in the group of animals treated per os with nimesulide or DMSO ## P<0.01 DMBA treated mammary glands from the group of animals treated with nimesulide versus DMSO.

**
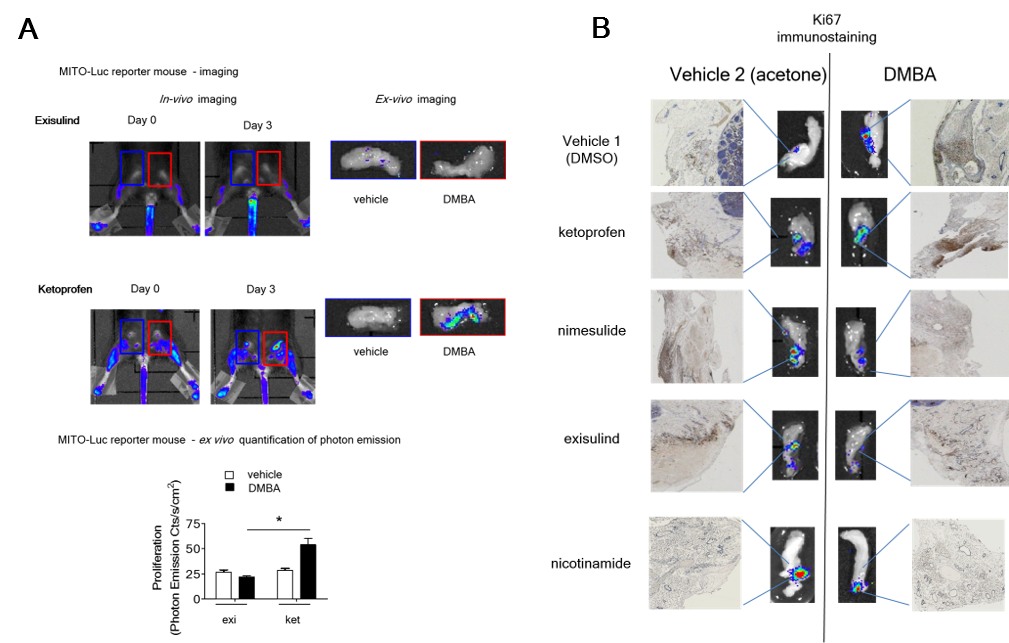
**

**Figure S5. Effects of NSAIDs, exisulind and nicotinamide on tissue proliferation.** (**A**) Pictures show the bioluminescence emission from the living repTOP*mito*IRE reporter mice (left set of pictures) or from the dissected mammary glands *ex vivo* (right set of pictures) of two representative animals of a group of five females treated with 15 mg/Kg of exisulind (exi) or ketoprofen (ket) (experimental scheme described in Figure S4B). Imaging was carried out before DMBA injection (day 0) and before sacrifice (day 3); after sacrifice, mammary glands were collected for *ex vivo* imaging. The graph reports the *ex vivo* quantification of the proliferative activity (photon emission) in the mammary glands; bars in the graphs are the average values of photon emission normalized over the area of the acquisition surface (cts/s/cm^2^), **P*<0.05 DMBA treated mammary glands of animals treated with ketoprofen *versus* DMBA treated mammary glands of animals treated with exisulind. (**B**) The bioluminescent signals reported in (**A**) and in Figure 4 correlate with the Ki-67 immunostaining in the tissue of repTOP*mito*IRE reporter mice. Pictures show the correlation between the bioluminescence emission and Ki67 staining in the same mammary glands (central and external set of pictures, respectively). After *ex vivo* imaging, mammary fat pads were fixed in 10% neutral buffered formalin and embedded in paraffin blocks. Four µm thick sections obtained from these blocks were then immunostained with a primary rabbit monoclonal antibody against Ki-67 antigen.

**Table S1. IC50 calculated from the *in vitro* assay.**

| **Molecules** | **IC50** | **Ki** |
| --- | --- | --- |
| Nicotinamide | 374 | 51 |
| Sulindac | 969 | 162 |
| Sulindac Sulphide | 376 | 66 |
| Exisulind | 397 | 62 |
| NS-398 | 862 | 144 |
| Nimesulide | 344 | 57.3 |
| Ketoprofen | no activity | no activity |
| Ibuprofene | 2152 | 359 |
| Ketorolac | 602 | 100 |
| Diclofenac | 554 | 92 |

| **ID** | **Treatment before surgery** | **Grade** | **Type** | **Disease state** | **pT** | **pN** | **ERα%** | **PgR%** | **HER2 score** | **HER2 %** | **Ki67 %** | **Age** |
| --- | --- | --- | --- | --- | --- | --- | --- | --- | --- | --- | --- | --- |
| 1 | Control | 2 | Lobular carcinoma, NOS | Primary | 1c | 0(sn) | 95 | 95 | 1+ | 80 | 19 | 73 |
| 2 | Control | 3 | Infiltrating duct and lobular carcinoma | Primary | 2 | 1a(sn) | 60 | 30 | 2+ | 90 | 26 | 50 |
| 3 | Control | 2 | Infiltrating duct carcinoma | Primary | 2(m) | 1 | 95 | 95 | Neg | n.d. | 18 | 61 |
| 4 | Control | 2 | Lobular carcinoma, NOS | Primary | 2(m,is) | 1a | 95 | 95 | 2+ | 20 | 21 | 58 |
| 5 | Ketorolac | 2 | Lobular carcinoma, NOS | Primary | 1c(m) | 0(sn) | 95 | 80 | Neg | n.d | 14 | 56 |
| 6 | Ketorolac | 1 | Lobular carcinoma, NOS | Primary | 1c(m) | 0(sn) | 90 | 85 | 1+ | 15 | 8 | 45 |
| 7 | Ketorolac | 2 | Infiltrating duct carcinoma | Primary | 1b(m) | 0(sn) | 95 | 25 | Neg | 0 | 21 | 61 |
| 8 | Control | 3 | Infiltrating duct carcinoma | Primary | 1c | 0(sn) | 15 | 15 | 3+ | 95 | 60 | 47 |
| 9 | Control | 3 | Infiltrating duct and lobular carcinoma | Primary | 2 | 1a | 95 | 95 | Neg | n.d. | 21 | 44 |
| 10 | Control | 2 | Infiltrating duct and lobular carcinoma | Primary | 3(is) | 1a | 95 | 95 | Neg | n.d. | 18 | 54 |
| 11 | Ketorolac | 3 | Infiltrating duct and lobular carcinoma | Primary | 2 | 3a | 95 | 80 | 2+ | 20 | 26 | 60 |
| 12 | Ketorolac | 3 | Infiltrating duct carcinoma | Contralateral | 1c | 0(sn) | 90 | 50 | 2+ | 40 | 24 | 72 |
| 13 | Ketorolac | 2 | Infiltrating duct carcinoma | Primary | 2 | 3a | 95 | 95 | Neg | n.d. | 22 | 45 |

**Table S2.** **Patients: diagnosis, treatments and tumour features**. n.d. not detected.
